# The role of miR‐29 family in disease

**DOI:** 10.1002/jcb.29896

**Published:** 2021-02-02

**Authors:** Masahiro Horita, Colin Farquharson, Louise A Stephen

**Affiliations:** ^1^ The Roslin Institute and Royal (Dick) School of Veterinary Studies The University of Edinburgh Midlothian Scotland UK

**Keywords:** cardiorenal disease, immune disease, microRNA, miRNA‐29, osteoarthritis, osteoporosis

## Abstract

MicroRNAs are small noncoding RNAs that can bind to the target sites in the 3’‐untranslated region of messenger RNA to regulate posttranscriptional gene expression. Increasing evidence has identified the miR‐29 family, consisting of miR‐29a, miR‐29b‐1, miR‐29b‐2, and miR‐29c, as key regulators of a number of biological processes. Moreover, their abnormal expression contributes to the etiology of numerous diseases. In the current review, we aimed to summarize the differential expression patterns and functional roles of the miR‐29 family in the etiology of diseases including osteoarthritis, osteoporosis, cardiorenal, and immune disease. Furthermore, we highlight the therapeutic potential of targeting members of miR‐29 family in these diseases. We present miR‐29s as promoters of osteoblast differentiation and apoptosis but suppressors of chondrogenic and osteoclast differentiation, fibrosis, and T cell differentiation, with clear avenues for therapeutic manipulation. Further research will be crucial to identify the precise mechanism of miR‐29 family in these diseases and their full potential in therapeutics.

## Graphical abstract

In the current review, we aimed to summarize the differential expression patterns and functional roles of the miR‐29 family in the etiology of diseases including osteoarthritis, osteoporosis, cardiorenal and immune disease. We present miR‐29s as promoters of osteoblast differentiation and apoptosis but suppressors of chondrogenic and osteoclast differentiation, fibrosis, and T cell differentiation.

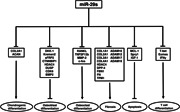

## 1 INTRODUCTION

MicroRNAs (miRNAs) are short, endogenous, single‐stranded noncoding RNAs of approximately 22 nucleotides in length, initially discovered in *Lin‐4* in the nematode *Caenorhabditis elegans*.^1^, ^2^ Since this initial discovery, miRNAs have been identified in plants, viruses, and animals including humans.^3^, ^4^, ^5^

**Figure 1:**
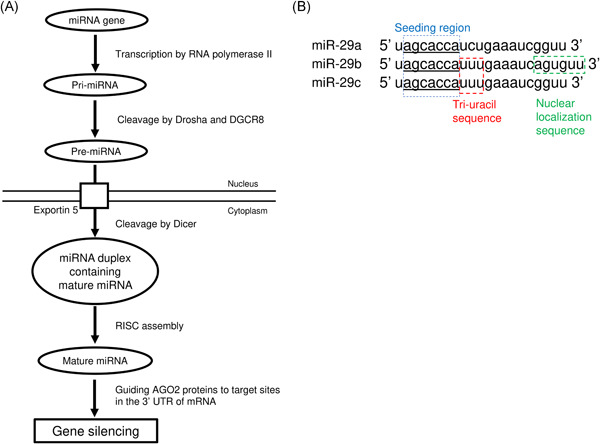
Maturation and function of microRNA (miRNA) and mature sequences of miR‐29s. (A) miRNA is transcribed by RNA polymerase II to generate a primary miRNA (pri‐miRNA), which is cleaved in Dorsha and DGCR8 to generate the precursor miRNA (pre‐miRNA). Pre‐miRNA is exported to the cytoplasm and then cleaved by Dicer to generate a miRNA duplex containing mature miRNA. The duplex unwinds and the mature miRNA assembles into RISC. Mature miRNA mediates gene silencing by guiding AGO2 proteins to target sites in the 3ʹ‐UTR of mRNA. (B) miR‐29 family members have identical seeding regions (blue box and underlined). Tri‐uracil nucleotide at positions 9‐11 exist in miR‐29b and miR‐29c (red box). Nuclear localization sequence at positions 18‐23 is unique to miR‐29b (green box). 3ʹ‐UTR, 3ʹ‐untranslated region; mRNA, messenger RNA

Transcription of miRNA generates a primary miRNA (pri‐miRNA), which is cleaved by the RNase III enzyme Drosha and the DGCR8 microprocessor complex subunit (known as Pasha in the model organisms *Drosophila melanogaster* and *Caenorhabditis elegans*).^6^, ^7^ This precursor (pre‐miRNA) is exported to the cytoplasm^8^ before cleavage by the RNase III enzyme Dicer, generating a miRNA duplex containing mature miRNA.^9^ The duplex unwinds and the mature miRNA assembles into RNA‐induced silencing complex (RISC).^10^, ^11^, ^12^ One strand of the mature miRNA (the “guide” strand) is loaded into Argonaute 2 (AGO2), whilst the “passenger” strand is degraded.^13^, ^14^ Mature miRNA guides AGO2 from the RISC complex to target sites in the 3ʹ‐untranslated region (3ʹ‐UTR) of messenger RNA (mRNA), inducing gene silencing (Figure 1A).^15^, ^16^, ^17^

miRNAs influence essentially all developmental processes and disease because miRNAs have conserved interactions with most human mRNAs.^18^ Aberrant levels of miRNA expression are found in many diseases where they adversely regulate posttranscriptional gene expression through transcriptional repression and/or degradation of target mRNAs, altering many cellular processes from proliferation and differentiation to apoptosis.^3^, ^19^, ^20^, ^21^, ^22^, ^23^ Therefore, understanding miRNA expression profiles in diseased tissues can guide diagnosis, prognosis, and prediction of therapeutic response.

The miR‐29 family are among the more commonly implicated miRNAs in disease. The miR‐29 family consist of miR‐29a, miR‐29b‐1, miR‐29b‐2, and miR‐29c, generated from two primary transcripts: pri‐miR‐29a/b1 cluster and pri‐miR‐29b2/c cluster, located on chromosomes 7q32.3 and 1q32.2, respectively, in humans. Although the sequences of pre‐miR‐29b1 and ‐2 are different, mature miR‐29b generated from both are identical.^24^, ^25^ Whilst miR‐29s are broadly conserved within mammals, miR‐29a is the most abundantly expressed family member.^26^, ^27^ The miR‐29s are identical at nucleotide position 2‐8, the seeding region that plays a role in recognizing the target mRNA (Figure 1B).^28^ Despite similar sequences, the miR‐29s have different subcellular localization with miR‐29a mainly located in the cytoplasm whereas miR‐29b and miR‐29c are concentrated in the nucleus.^28^, ^29^ miR‐29b has a unique six nucleotide segment, which leads to its nuclear localization.^28^ This nuclear localization contributes to chromosomal segregation and nuclear morphology through unconventional mechanisms not traditional to mRNA targeting.^30^ Both miR‐29b and miR‐29c have a tri‐uracil residue at positions 9–11, leading to rapid decay or turnover, whereas the cytosine residue at nucleotide position 10 of miR‐29a contributes to its stability.^31^

Many miRNAs are abnormally expressed in disorders as diverse as osteoarthritis (OA), osteoporosis, cardiorenal disease, and immune disease. The miR‐29 family however is central to the etiology and pathogenesis of these diseases.^32^, ^33^, ^34^, ^35^, ^36^ In this review, we will explore the expression, regulation, and function of miR‐29 family members, providing fundamental insight into their critical role in the pathogenesis of several debilitating diseases that are of great public health concern.

## 2 ROLE OF MIR‐29 FAMILY IN OSTEOARTHRITIS

OA is a degenerative joint disease, mostly of the elderly, involving degradation of articular cartilage, subchondral bone sclerosis, chondro‐osteophyte formation, and inflammation of the joint.^37^ The etiology of OA is not fully recognized and there are currently no effective treatments for OA aside form pain control, physiotherapy and finally, prosthetic joint replacement in the most severe cases.^38^

Articular chondrocytes are generated through the process of chondrogenesis, which begins with the condensation of mesenchymal stem cells (MSCs).^39^ MSC‐directed chondrogenic differentiation is an attractive OA treatment target for cartilage repair and regeneration, and Dicer, essential in generation of mature miRNAs, has a critical role in controlling chondrocyte cell proliferation and differentiation.^40^, ^41^, ^42^ miR‐29s are critical for controlling chondrogenic differentiation of MSCs, the downregulation of miR‐29a/b expression in mature chondrocytes compared to MSCs, suggests that high levels of expression may impair development of the mature chondrocyte phenotype.^32^, ^43^, ^44^, ^45^ Indeed, miR‐29a overexpression inhibits the expression of chondrocyte‐specific markers, such as Type II collagen and aggrecan, during chondrogenic differentiation, whereas decreased expression of miR‐29a/b, which directly targets the 3ʹ‐UTR of Col2a1, is necessary for human chondrogenic differentiation.^44^, ^45^ Downregulation of miR‐29a/b is controlled by the transcription factor, Sry‐related box 9 (Sox9) in both human and murine cells. Furthermore, increased Sox9 expression is essential for the formation of MSC condensations.^32^, ^44^, ^46^, ^47^, ^48^

Transforming growth factor‐β (TGF‐β) signaling is also critical for the initiation of chondrogenesis.^49^, ^50^ Phosphorylation of Smad2 and Smad3 by TGF‐β leads them to form a heteromeric complex in association with Smad4.^51^, ^52^ These Smad complexes move into the nucleus and inhibit hypertrophic chondrocyte differentiation.^53^ Human OA chondrocytes have been used to show that TGF‐β1 ligand, as well as phosphorylated Smad2/3, downregulate miR‐29a/b/c expression, suggesting miR‐29s in collaboration with the TGF‐β1signalling pathway, may play a role in the etiology of OA.^32^ Furthermore, miR‐29b overexpression inhibits TGF‐β1 induced Smad2/3/4 signaling, whilst inhibition of miR‐29b augments the TGF‐β1 response.^32^ As well as controlling TGF‐β1 ability to modulate Smad2/3/4 signaling, the downregulation of miR‐29a causes synovial fibroblasts to increase TGF‐β1 expression, whereas miR‐29a overexpression results in decreased TGF‐β1 expression.^54^ In summary, the impact of miR‐29s on TGF‐β1‐induced Smad signaling in the etiology of OA warrants further study, which could lead to an attractive treatment strategy for cartilage repair and regeneration in OA.

Many reports have described the expression profiles of miR‐29s in preclinical OA animal models and human OA tissues; miR‐29a is downregulated and miR‐29b is upregulated in OA cartilage compared with healthy cartilage samples.^32^, ^55^, ^56^ OA patients have higher expression of miR‐29c in their plasma and similarly miR‐29c levels in synovial fluid are positively associated with the severity of knee OA.^57^, ^58^ In mouse models, miR‐29b expression peaks in the days following destabilization of the medial meniscus surgery, but decreases gradually with advancing OA to reach equivalent levels to controls within 6 weeks.^32^, ^55^ How miR‐29s contribute to cartilage destruction in OA is unclear but their involvement may be linked to the actions of proinflammatory cytokines, such as interleukin‐1 β (IL‐1β) and tumor necrosis factor alpha (TNF‐α), that are released from the synovium and chondrocytes during OA pathogenesis.^59^, ^60^ IL‐1β has been reported to increase or decrease the expression of miR‐29b in human OA chondrocytes in culture whereas IL‐1β stimulation of the chondrosarcoma SW1353 cell line has no effect on miR‐29b expression.^32^, ^61^, ^62^ There are fewer studies reporting the effects of TNF‐α on miR‐29s in OA but in one study, SW1353 chondrocytes were stimulated with TNF‐α to mimic OA transcriptional reprogramming and miR‐29b expression was down regulated after 24 h exposure.^61^ In OA, increased levels of proinflammatory cytokines released by chondrocytes and synovium leads to increased levels of matrix metalloproteinase (MMPs) and the degradation of ECM proteins, such as aggrecan and collagen Type II.^63^, ^64^ The ability of miR‐29s to mediate the pro‐inflammatory response and alter MMP expression has been the focus of various studies but to date there is little consensus on whether miR‐29s can promote or inhibit MMP expression. miR‐29b repression of MMP‐3 expression has been noted in IL‐1β challenged human and murine chondrocytes as well as miR‐29a restoration of cartilage deteriorationin a in vivo collagenase induced OA model.^32^, ^54^ In the latter study, undamaged (smooth) articular cartilage was observed, and MMP‐13 expression was reduced in OA joints of miR‐29a transgenic mice compared to wild type mice.^54^ Whilst these data suggest that miR‐29a can protect articular cartilage by down regulating MMP‐13, conflicting studies indicate that miR‐29b overexpression can lead to increased expression of MMP1, 13, and Type X collagen and decreased expression of Type II collagen in both rat primary chondrocytes and SW1353 cells.^54^, ^55^ Similarly, in a rat OA model the injection of a miR‐29b antagomir inhibited the production of MMP1, 13, and type X collagen, whilst protecting Type II collagen and aggrecan from degradation.^55^ The proinflammatory cytokine, IL‐1β can also activate nuclear factor‐kappa B (NF‐κB) signaling during the progression of OA, interestingly miR‐29b has been reported to repress NF‐κB signaling and reduce articular cartilage catabolic effects. The effects of miR‐29b on NF‐κB signaling have also been shown in SW1353 chondrocytes where a miR‐29b inhibitor upregulated IL‐1β‐induced activation of NF‐κB signaling.^32^, ^65^

Although numerous other cytokines, such as IL‐6, IL‐15, IL‐17, IL‐18, and IL‐21 are associated with OA pathogenesis, their effect, if any, on miR‐29 expression in healthy human chondrocytes and synovial tissues remain unclear.^59^ For example, IL‐4 is widely recognized as an anabolic cytokine with an ability to inhibit IL‐1β‐induced release of MMP‐13 in OA chondrocytes.^66^ As IL‐4 inhibits miR‐29a expression in systemic sclerosis fibroblasts it is possible that a complex interplay between different cytokines and the expression of miR‐29s exist to control MMP production and cartilage degradation in OA.^67^ Nevertheless, the precise mechanisms remain unclear and further in vivo and in vitro studies with primary cells are required.

Other signaling pathways associated with OA pathogenesis, such as Wnt/β‐catenin signaling may also involve miR‐29s. Wnt/β‐catenin signaling induces hypertrophic differentiation of chondrocytes which leads to the degeneration of articular cartilage and progression of OA.^68^, ^69^ Whilst only a few studies have been reported, the miR‐29s can negatively regulate Wnt/β‐catenin signaling through the inhibition of Frizzled receptors FZD3, FZD5, and Disheveled 3, which are required for Wnt activation.^32^, ^70^, ^71^ Wnt/β‐catenin signaling is regulated via extracellular inhibitors, such as Dickkopf‐1 (DKK‐1) and sclerostin.^72^, ^73^ DKK‐1 prevents the destruction of articular cartilage, subchondral bone sclerosis and chondro‐osteophyte formation, whereas sclerostin deficiency promotes OA in mice via subchondral bone sclerosis.^74^, ^75^ Whilst this is robust evidence that inhibitors of Wnt/β‐catenin signaling can prevent OA, the precise role of miR‐29s in controlling DKK‐1 and SOST is unknown but further investigations could identify a potential therapeutic target for OA.

## 3 ROLE OF MIR‐29 FAMILY IN OSTEOPOROSIS

Osteoporosis is an asymptomatic, chronic, and degenerative bone disease resulting in an increased risk of bone fracture and reduced life quality.^76^, ^77^, ^78^, ^79^ In osteoporosis a dysregulation of osteoblast and osteoclast differentiation and activity results in an imbalance between bone formation and resorption during bone remodeling. This results in structural changes to both trabecular and cortical bone and a reduced bone mineral density (BMD).^80^, ^81^, ^82^ Osteoblasts and osteoclasts are derived from different embryonic cell lineages; the former differentiate from MSCs whereas the latter derive from hematopoietic progenitors in the monocyte or macrophage lineage.^83^, ^84^, ^85^ Mature osteoblasts synthesize bone matrix and terminally differentiate into osteocytes when embedded in mineralizing osteoid. Osteocytes maintain structural bone integrity and allow bone to adapt to mechanical stimuli.^80^, ^86^ Osteoporosis is most common in post‐menopausal women where reduced circulating estrogen levels lead to an increase in both bone resorption and bone formation. However, the increase in bone resorption predominates leading to a net bone loss.^87^, ^88^, ^89^

To date, therapeutic agents, such as anti‐resorptive and anabolic drugs are the preferred treatment for osteoporosis. Anti‐resorptive drugs, such as bisphosphonates and anti‐RANKL treatment, for example, denosumab function by inhibiting the recruitment and activity of osteoclasts, leading to a decreased bone remodeling rate. Anabolic drugs, such as 1‐34 parathyroid hormone treatment, (e.g., teriparatide), and anti‐sclerostin, (e.g., romosozumab) by contrast, lead to increased bone formation via a positive remodeling balance.^90^ Regardless of the ability of treatment options, the therapeutic options for osteoporosis aren't sufficient for many patients with a high risk of fractures and romosozumab has recently been associated with adverse cardiovascularevents.^90^, ^91^ It is therefore appropriate for other pathways involved in bone formation and resorption to be considered as therapeutic targets in the quest to maintain or improve bone mass in osteoporosis patients.

Possible new diagnostic biomarkers or targets for pharmaceutical intervention include miRNAs and specifically miR‐29s. In preclinical studies, miR‐29a expression in bone of ovariectomised mice are decreased and in human studies serum levels of miR‐29a levels are lower in postmenopausal women with low BMD.^33^, ^92^ Serum levels of miR‐29b are also lower in patients with bone fragility fractures and are positively correlated with both histomorphometric parameters of bone formation and circulating levels of procollagen Type 1 N‐terminal propeptide; a recognized biomarker of bone formation.^93^, ^94^, ^95^ Whilst there is a paucity of data reporting the circulating levels of miR‐29s in osteoporosis, there are numerous studies reporting the essential role of miR‐29s for osteoblast and osteoclast differentiation and function during the bone remodeling process.^80^

Osteoblast differentiation is a coordinated process that is tightly regulated by growth factors, transcription factors and intracellular signaling pathways.^96^, ^97^, ^98^, ^99^, ^100^, ^101^, ^102^ In particular the Wnt/β‐catenin and TGF‐β/bone morphogenetic protein (TGF‐β/BMP) signaling pathways, which have fundamental roles in skeletal development and bone homeostasis. Canonical Wnt signaling requires the phosphorylation and stabilization of cytoplasmic β‐catenin to upregulate the expression of runt‐related transcription factor 2 (Runx2) and the promotion of osteoblast differentiation.^99^ Alternative signaling cascades are initiated by TGF‐β/BMP signaling via Smad proteins or noncanonical, non‐Smad pathways. These include various branches of mitogen‐activated protein kinase (MAPK) pathways, such as extracellular signal–regulated kinase (ERK), c‐Jun N‐terminal kinase (JNK), and p38 MAPK (p38).^96^, ^97^, ^98^, ^100^, ^101^, ^102^ In contrast to Wnt/β‐catenin signaling, TGF‐β signaling suppresses the later phase of osteoblast differentiation and matrix mineralization through a reduction of Runx2 expression.^103^ Similarly, the class IIa histone deacetylases, HDAC4 and ‐5 are widely expressed in MSCs^104^ and can lead to the degradation of Runx2 and impairment of osteoblast differentiation.^102^, ^105^ They also act as corepressors for TGFβ/Smad3‐mediated transcriptional repression of Runx2 function in osteoblast differentiation.^106^ Intriguingly, the expression of miR‐29s increase during osteoblast differentiation, a role consistent with the observation that miR‐29a/b can reduce HDAC4 and induce Runx2 and Wnt/β‐catenin signaling during osteoblast differentiation^33^, ^107^, ^108^, ^109^, ^110^, ^111^ miR‐29a can promote osteoblast differentiation by other routes including the negative regulation of CTNNBIP1 (inhibitor of WNT/β‐catenin signaling), DUSP2 (inhibitor of JNK‐MAPK pathway) and CDK6 (BMP2 antagonist) and ACVR2A, a recognized inhibitor of osteoblast differentiation.^109^, ^111^, ^112^ Furthermore, miR‐29a can also promote osteoblast differentiation by activating Wnt/β‐catenin signaling through the ERK‐MAPK pathway and via a positive feed forward loop in which canonical Wnt signaling induces miR‐29a transcription resulting in the down regulation of antagonists of β‐catenin‐dependent Wnt signaling, DKK1, Kremen2, and secreted fizzled related protein 2. This cascade of events will potentiate Wnt signaling, driving a gene expression program essential for osteoblast differentiation.^107^, ^113^

The principal function of the fully differentiated osteoblast is to synthesize and excrete a collagen rich ECM in bone (osteoid) which is mineralized through actions of the osteoblast over time. miR‐29a/c negatively regulates the expression of a number of ECM genes and accordingly the mineralization stage of osteoblast differentiation is characterized by decreased ECM gene expression and increased expression of miR‐29a/c.^108^, ^114^, ^115^ For example, miR‐29a/c inhibits the expression of osteonectin or secreted protein acidic and rich in cysteine, which is a collagen‐binding matricellular protein and critical for ECM assembly and deposition.^108^ miR‐29b also suppresses the production of collagens Type I, IV, V by the differentiated osteoblast and during ECM mineralization.^111^, ^116^ The overexpression of miR‐29a in fish bone‐derived cells can lead to increased expression levels of BMP2, osteocalcin, and osteopontin to accelerate differentiation and induce ECM mineralization, but the impact of miR‐29a on ECM mineralization is attenuated at the end stage of the differentiation.^117^ These results suggest that miR‐29s are positive regulators for osteoblast differentiation and ECM mineralization, whilst miR‐29s could function as negative regulators to avoid excessive ECM accumulation during skeletal mineralization.

Osteoclast differentiation from hematopoietic precursors is driven by macrophage colony‐stimulating factor and receptor activator for nuclear factor κB ligand (RANKL), which are expressed by osteoblasts, osteocytes and activated T cells.^118^, ^119^ RANKL activates its receptor, RANK on osteoclast precursors to promote a signaling cascade involving many transcriptional factors, including c‐fos, NF‐κB (p50 and p52), JNK, p38 MAPK, TRAF6, and NFATc1, resulting in osteoclast differentiation and bone resorption.^120^, ^121^, ^122^, ^123^, ^124^, ^125^ The RANK/RANKL interaction together with the soluble decoy receptor osteoprotegerin (OPG) is widely recognized to control osteoclast differentiation and bone resorption.^126^, ^127^ Osteoclast differentiation is enhanced by proinflammatory cytokines, such as TNF‐α, IL‐1β, IL‐6 and IL‐17, whereas anti‐inflammatory cytokines, such as IL‐4, IL‐12, IL‐33 and interferons inhibit their differentiation.^118^, ^119^, ^128^

Dicer, DGCR8, and AGO2, essential components in the generation of mature miRNAs, have critical roles in osteoclast differentiation.^129^ Specifically, the expression of miR‐29a by osteoclast precursors is stimulated by TNF‐α; an observation consistent with other studies reporting that in murine osteoclast precursors and the mouse monocyte cell line, RAW264.7, the expression of miR‐29s are increased during RANKL‐induced osteoclast differentiation, in concert with osteoclast markers Trap and cathepsin K.^130^, ^131^ In addition, miR‐29 knockdown led to a delay in osteoclast differentiation and the migration of osteoclast precursors but did not affect actin ring formation by mature osteoclasts.^131^ Identification of target genes for miR‐29s during osteoclast formation specified that miR‐29s promoted osteoclast formation by targeting RNAs important for cytoskeletal organization, commitment, and osteoclast function.^131^ Moreover, miR‐29b can increase osteoclast survival rate by repressing the proapoptotic gene, BCL‐2‐modifying factor.^132^ Whilst these data imply that miR‐29s are positive regulators of osteoclast differentiation a conflicting study reported that miR‐29b functions as a negative regulator of human osteoclast differentiation and bone resorption by downregulating the expression of c‐fos and MMP2.^133^ This negative regulation of osteoclast formation and function by miR‐29b was recently corroborated in an in vitro study that disclosed upregulation of osteoclast differentiation and pit formation when miR‐29a expression was silenced.^33^ Similarly, mice overexpressing miR‐29a in osteoblasts had increased bone mass and estrogen deficiency‐induced bone loss was mitigated in miR‐29a overexpressing mice. Mechanistically, it was found that miR‐29a signaling in osteoblasts is bone protective through repression of osteoclast regulators, RANKL and CXCL12, to reduce osteoclastogenic differentiation.^33^ Different culturing conditions may explain the reported discrepancies in the ability of miR‐29s to modify osteoclast formation and activity, but the in vivo data from Lian and colleagues provides compelling insight into the complex interplay between osteoclasts and osteoblasts, revealing the remedial potential of miR‐29a for improving osteoporotic disorders.

Glucocorticoids (GCs) are prescribed for the treatment of many chronic conditions, but their chronic use is associated with frequent and wide‐ranging adverse effects including osteoporosis and bone fractures.^134^ These adverse bone effects are also observed in Cushing's disease which is caused by increased secretion of adrenocorticotropic hormone from the pituitary gland that stimulates the synthesis of cortisol by the adrenal glands leading to accelerated bone loss in both men and women.^135^ The precise mechanisms that underlie the undesirable effects of GCs on skeletal development are unclear but are likely to involve impairment of both osteoblast and osteoclast differentiation and function. However, the ability of GCs to promote osteoclast formation and activity by increasing RANKL production by both osteoblasts and osteocytes and downregulating its soluble decoy receptor OPG is the prevailing mechanism. This skews the RANKL: OPG ratio towards osteoclastogenesis.^136^ Interestingly, miR‐29s have also been reported to mediate the catabolic effects of GCs on the skeleton. GC treatment reduces miR‐29a but not miR‐29b or miR‐29c expression in rat bone and miR‐29a overexpression promotes *Runx2* expression and reduces the ability of GCs to inhibit osteoblast differentiation, BMD, and trabecular bone volume.^113^ miR‐29a overexpression in mice weakened GCs ability to promote RANKL expression, osteoclast differentiation, and bone erosion, independent of altered OPG expression.^110^, ^137^ The mechanisms by which miR‐29a protects the skeleton from GCs may involve miR‐29a inhibiting GC‐induced DKK‐1 expression, which would result in increased Wnt/β‐catenin signaling and osteoblast differentiation.^113^ Alternatively, miR‐29a may delay or inhibit GC induced bone resorption by repressing tumor necrosis factor superfamily 13b expression, which supports osteoclast differentiation and maturation.^137^ In conclusion, the consensus across a variety of published studies is that miR‐29s inhibit osteoclast differentiation; this may make it an attractive target to reduce bone resorption in osteoporosis and other skeletal disorders with excessive osteoclastic bone resorption.

## 4 ROLE OF MIR‐29 FAMILY IN CARDIORENAL SYNDROME

Cardiorenal syndrome is a complex and severe clinical condition defined as a pathophysiological disorder of the heart and kidneys whereby acute or chronic dysfunction in one organ induces the same in the other. This ultimately leads to both chronic heart failure (CHF) and chronic kidney disease (CKD).^138^, ^139^ A common pathological feature of cardiorenal syndrome is fibrosis and in particular an excessive accumulation of collagen and fibronectin within the ECM.^140^, ^141^ Fibrosis is a common consequence of inflammation‐ and oxidative stress‐ related endothelial dysfunction in aging, hypertension, diabetes mellitus (DM), obesity, ischemia, and organ injury.^142^ However, the cellular mechanisms leading to fibrosis are unclear and therefore current management of patients with CRS are mainly via supportive therapies to relieve the progression of the diseases.^142^, ^143^ Cardiac myofibroblasts, the main cell type in the heart, are derived from multiple cell lineages, including resident fibroblasts, smooth muscle cells, epithelial and endothelial cells via epithelial or endothelial‐mesenchymal transition (EMT/EndMT), and fibrocytes.^144^ When cardiac myofibroblasts synthesize excess collagen and other ECM proteins, the resultant fibrosis can lead to myocardial infarction, cardiomyopathy, and heart failure.^145^, ^146^, ^147^, ^148^

Dicer has a critical role in the development of the ventricular myocardium and the preservation of glomerular and podocyte function and its deletion, unsurprisingly leads to cardiac hypertrophy, ventricular fibrosis, glomerulosclerosis, tubulointerstitial fibrosis, podocyte foot process effacement, and proteinuria.^149^, ^150^, ^151^, ^152^, ^153^ The heart and kidney have high expression levels of miR‐29s which are lower in animal models and human samples of CHD and CKD possibly implicating miR‐29s as protective agents in these two organs.^154^, ^155^, ^156^, ^157^, ^158^ Similarly, the expression of miR‐29s in cardiac muscle of mice and humans are also downregulated in the region of a myocardial infarction in mice and humans, which may account for the increased expression of collagen Types I, II, III, and fibrillin 1.^156^ In support of this protective role, miR‐29a expression is upregulated in the heart and cardiac fibroblasts of aged zebrafish which may prevent collagen deposition and DNA methylation through the inhibition of DNA methyltransferase, such as DNMT1 and DNMT3a to avoid cardiac damage.^159^ Indeed, fibrosis and DNA methylation in human cardiac fibroblasts is a consequence of hypoxia downregulating miR‐29a/b expression.^159^

In atrial fibrosis, miR‐29s expression is lower in a canine model of congestive heart failure‐related atrial fibrillation (AF). Similarly, the expression of miR‐29b in both serum and atrial tissues are decreased in humans with congestive heart failure and/or AF whereas overexpression of miR‐29b in canine or murine fibroblasts results in decreased expression of collagen Types I, lll, V, and fibrillin 1.^160^, ^161^ miR‐29a can also inhibit cardiomyocyte hypertrophy by the inhibition of nuclear factor of activated T cells c4 (NFATc4)and/or the suppression of peroxisome proliferator‐activated receptor δ.^162^, ^163^ These results together strongly support a protective role for tissue miR‐29s against the development of cardiac hypertrophy and fibrosis. In contrast, the role of circulating miR‐29s is less certain as levels are upregulated in patients with essential hypertension and hypertrophic cardiomyopathy and these levels are positively correlated with left ventricular hypertrophy and myocardial fibrosis.^164^, ^165^, ^166^ Various rodent models have also indicated that the tissue levels of miR‐29s are not protective against transverse aortic constriction (TAC)‐induced cardiac hypertrophy. Increased expression of miR‐29a in cardiac tissue is associated with TAC‐induced cardiac hypertrophy whereas miR‐29 deficient mice or those treated with miR‐29a antagomirs had reduced levels of TAC‐induced cardiac hypertrophy and myocardial fibrosis.^164^, ^166^, ^168^ miR‐29a expression is also upregulated in murine heart tissues of myocardial ischemia‐reperfusion injury and overexpression of miR‐29a promotes cell apoptosis through suppression of insulin‐like growth factor I in a rat myoblast cell line^169^ Moreover, suppression of miR‐29a/c can reduce myocardial infarct size and IR injury‐induced cell apoptosis via the upregulation of myeloid cell leukemia 1 (MCL‐1), which is a target of miR‐29s.^170^ In summary, the precise roles of miR‐29s on cardiac disease are still unclear as the miR‐29s levels of the tissues appear to be protective for myocardial infarction, congestive heart failure, and AF in some reports whilst conflicting studies indicate that miR‐29s could function as a progressive factor for TAC‐induced cardiac hypertrophy and myocardial IR injury.

Renal myofibroblasts originate from various origins, including bone marrow‐derived fibroblasts, tubular epithelial cells, endothelial cells, pericytes and interstitial fibroblasts and are responsible for any excess matrix production in renal fibrosis.^171^, ^172^ TGF‐β signaling is a recognized central mediator of renal fibrosis, possibly through its ability to inhibit miR‐29s capacity to suppress the deposition of collagen Types I, III, and IV by mesangial cells, tubular cells and podocytes in both humans and rodent models.^155^, ^173^, ^174^, ^175^ A disintegrin and metalloproteinases (ADAMs) are involved in renal fibrosis and TGF‐β/Smad2/3 signaling upregulates Adam 10, 12, 17, 19 expression in renal cells and in unilateral ureteral obstruction models of renal fibrosis.^35^ The increase in Adams12 and 19 expression correlated strongly with a decrease in miR‐29s expression and the overexpression of miR‐29s blocked TGF‐β‐mediated upregulation of Adam12 and Adam19 gene expression and improve renal fibrosis.^35^, ^36^, ^176^ These studies strongly suggest ADAMs are involved in renal fibrosis and are regulated by both miR‐29s and TGF‐β making thempotential therapeutic targets for the prevention of renal fibrosis.

Patients with CKD present with cardiac fibrosis, hypertrophy, and dysfunction.^177^, ^178^ In a rat CKD model, increased levels of circulating cardiotonic steroids (CTS) and activation of Na/K‐ATPase induce cardiac fibrosis and hypertrophy.^179^ This in turn negatively regulates miR‐29b resulting in increased collagen Type I synthesis and fibrosis.^178^, ^180^ This key role for miR‐29b was confirmed in miR‐29b overexpression studies where CTS‐induced collagen Type I synthesis in rat cardiac fibroblasts was inhibited.^178^

Diabetic cardiomyopathy (DC) is characterized by myocardial fibrosis, the major cardiovascular complication in patients with DM.^181^ The serum levels of IL‐6 are elevated in patients with DM and are positively associated with one‐year mortality outcomes in patients with CHF.^182^, ^183^ Elevated serum IL‐6 levels in experimental diabetic mice promotes TGF‐β1 expression and the downregulation of miR‐29a. This results in the increased synthesis of collagen Types I and III, an effect that is inhibited by the overexpression of miR‐29a by cardiac fibroblasts.^184^ In contrast, the promotion of DM can induce miR‐29s expressions and inhibit MCL1 expression, which results in apoptosis of mouse cardiomyocytes.^185^ Insulin, which is used for the treatment of DM, inhibits miR‐29s via increased mammalian target of rapamycin complex 1 (mTORC1) signaling. Conversely, the suppression of mTORC1 signal pathway initiates upregulation of miR‐29s and downregulation of MCL‐1, resulting in the loss of myofibril bundle organization in rats with DM.^185^ These results suggest that the impact of DM on the expression and function of miR‐29s in DC may be different depending on the type of cells and experimental conditions studied.

In addition to the effects of DM on cardiac disease, diabetic nephropathy (DN), which is characterized by glomerulosclerosis and tubulointerstitial fibrosis, is the most common cause of CKD in patients with DM.^186^ Pathogenesis of DN is attributable to hyperglycemia‐induced TGF‐β/Smad signaling which leads to the fibrotic changes typical of DN.^187^, ^188^, ^189^ Serum and renal levels of miR‐29b are downregulated in patients with Type 2 DM and this leads to the promotion of collagen Types I, III, and IV expression and renal fibrosis in the diabetic mouse.^175^, ^190^, ^191^ Whilst miR‐29s are suppressed in kidneys of diabetic mice, dipeptidyl peptidase‐4 (DPP‐4) inhibitor; a therapeutic drug for Type 2 DM, can restore miR‐29s and potentially limit fibrosis.^159^ In addition, Wnt/β‐catenin signaling is impaired in glomeruli of DM‐induced mice whereas DKK‐1 and fibronectin expression are upregulated.^192^ This increased expression of DKK‐1 and fibronectin is a likely consequence of reduced levels of miR‐29a as both are normalized in glomeruli of miR‐29a transgenic DM mice.^192^ Moreover, overexpression of miR‐29a can rescue the high glucose‐induced cell apoptosis and upregulation of fibronectin in mouse mesangial cells maintained in vitro.^192^ Similarly, in murine podocytes and glomeruli of miR‐29a transgenic mice, high glucose‐induced deacetylation and ubiquitination of nephrin, which promotes podocyte apoptosis and renal fibrosis, are also inhibited via the suppression of HDAC4.^193^ In contrast to miR‐29a, miR‐29c is higher in high glucose‐treated murine podocytes and glomeruli of diabetic mice which leads to cell apoptosis and increases fibronectin synthesis through a coordinated coupling of Sprouty homolog 1 and Rho kinase.^194^, ^195^

Angiotensin II (Ang II) activates several intracellular signaling pathways, such as TGF‐β/Smads, NF‐κB, and IL‐6 to promote both cardiac and renal fibrosis and inflammation.^196^, ^197^, ^198^, ^199^ Smad7 is induced by activation of Smad2/3 and can suppress TGF‐β‐induced renal and cardiac fibrosis by blocking Smad2/3 phosphorylation via a negative feedback mechanism.^200^ Smad7 also plays a key role in suppressing renal inflammation by downregulating NF‐kB signaling.^187^, ^201^ The central role for Smad7 in CRS was demonstrated in a Smad7 deficiency model, which resulted in enhanced ANG II‐induced loss of miR‐29b expression and the promotion of murine cardiac and renal fibrosis through activation of TGF‐β/Smad3 and NF‐kB signaling.^202^, ^203^ Angiotensin‐converting enzyme inhibitors can effectively protect against renal fibrosis and reduce the incidence of CKD by inhibiting DPP‐4, phosphorylation of Smad3 and increasing miR‐29s expression in the kidneys of DM‐induced mice and in human endothelial cells.^204^, ^205^ Indeed, overexpression of miR‐29b can repress Ang II‐induced EMT through the inhibition of phosphoinositide 3‐kinase/AKT signaling, resulting in less renal interstitial fibrosis.^206^ Moreover, miR‐29b can also play a protective role in cardiac fibrosis by inhibiting Ang II‐mediated TGF‐β/Smad3 signaling.^207^ Whilst the available studies suggest that miR‐29s are essential negative regulators for cardiac and renal fibrosis the precise mechanistic roles of miR‐29s on both diseases remain to be clarified.

## 5 ROLE OF MIR‐29 FAMILY IN IMMUNE DISEASE

miRNAs are well known to play an important role in maintenance of the immune system.^34^, ^208^ This was first noted in Dicer‐deficient T cells, which exhibit a preference for Th1 polarization, but has since been shown at many stages of immunological function and development.^208^ It is worth noting that miR‐29s are expressed in both T cells and B cells, meaning they have the potential to influence a huge range of processes.^34^ Furthermore, miR‐29s are crucial for regulating an immune response to several viruses.^209^, ^210^, ^211^

Adaptive immunity relies on the production of T cells in the thymus, where, in response to antigen stimulation, the thymic epithelium induces naïve T cell production.^212^, ^213^ T cells will be directed down a T cell type fate, the most commonly studied being Th1 and Th2, important in regulating the response to either intracellular (Th1) or extracellular (Th2) assault.^213^ The miR‐29s are vital in both of these steps. Targeted deletion of miR‐29a/b, in vivo, phenocopies Dicer deficient cells, by reducing the threshold for thymic involution, subsequently suppressing T cell production.^34^, ^208^, ^214^ Chandiran and colleagues show that the initial direction of naïve cells down the Th1 cell fate is only possible through noncanonical Notch1‐mediated repression of miR‐29s.^214^, ^215^ Notch1 repression is attenuated later in Th1 differentiation, by the effects of IFNƳ; this releases the repression of miR‐29s, therefore repressing further production of Th1 cells.^214^ The pri‐miR‐29a/b1 cluster is in fact central to a negative regulatory feedback loop, required to maintain T cell balance.^216^ Acting through the IFNƳ pathway, miR‐29 suppresses the Th1 cell‐fate, required for cell‐mediated immunity.^216^, ^217^ miR‐29a/b directly inhibits the expression of the crucial T cell markers IFNƳ (inducer of miR‐29 expression), and T‐box binding transcription factor (T‐bet [inducer of IFNƳ]), through miR‐29 seed sequences in these genes. Treatment of miRNA‐deficient cells with either miR‐29a/b is sufficient to restore wild type levels of T‐bet and IFNƳ, while loss of miR‐29a/b results in unregulated production of Th1 cells as well as IFNƳ and T‐bet.^216^, ^217^

The importance of miR‐29a in immunological function and disease is in part due to the enrichment of miR‐29s and their targets in both T and B cells.^34^ Mice null for miR‐29c in particular exhibit reduced B cell response, and are protected against collagen‐induced arthritis.^218^ Repression of B cell miR‐29s expression via AKT and MYC pathways is associated with loss of apoptosis and several B cell malignancies, particularly lymphomas.^218^, ^219^, ^220^, ^221^ Likewise, the downregulation of miR‐29s in human immunodeficiency virus (HIV) patients is associated with higher incidence of B cell tumors via the MYC pathway.^223^ The role of miR‐29s in these major pathways means that miR‐29s directed therapeutics are being considered for non‐Hodgkin's lymphoma, myeloid leukemia, and aggressive B cell lymphoma as well as the autoimmune disease, Crohn's disease.^222^, ^223^, ^224^, ^225^, ^226^

The autoimmune disease, multiple sclerosis (MS) is associated with a significant increase in pro‐inflammatory Th1 cells, which have a de‐myelinating effect in the central nervous system.^227^ miR‐29ab1 deficiency is noted in both MS and the classic mouse model, experimental autoimmune encephalomyelitis.^216^ Ma and colleagues suggest that an increase in Th1 cells, as seen in MS patients, is linked to an increase in miR‐29b specifically, although Smith and colleagues posit that these differences are likely to be cell type and context‐specific, noting a specific increase in miR‐29b within memory cells of MS patients.^216^, ^228^ One of the first line treatments for MS is Interferon‐β (IFN‐β) treatment, as well as improving clinical symptoms of the disease; IFN‐β reduces inflammatory response and reduces expression of miR‐29s.^229^, ^230^ While many current treatments look to realigning the Th1/2 balance in MS patients, it is hoped that miR‐29s treatments may provide future therapeutic avenues.

Type 1 DM is an autoimmune disease, caused by a loss of function in the insulin‐secreting β cells of the pancreas. This loss of function follows an influx of proinflammatory cytokines into the pancreatic islets of Langerhans, destroying large numbers of β cells and reducing capacity to secrete insulin in response to rises in blood sugar.^231^ Interestingly miR‐29 is among the most highly expressed microRNA in healthy β cells.^232^ Experiments in both pre‐diabetic NOD mice and insulin‐impaired MIN6 cells suggest miR‐29s are highly upregulated in prediabetic β cells in direct response to cytokine action. In fact, over expression of miR‐29s caused a diabetes‐like reduction in insulin secretion in both human and mouse islet cells and directly suppresses antiapoptotic gene, MCL1.^233^ miR‐29s are considered key markers of diabetes and prediabetes and certainly warrants further study to fully understand its potential in furthering our understanding of the disease and potential therapies.^233^, ^234^, ^235^, ^236^

The role of the miR‐29s in HIV infections is an emerging but exciting field. Since miRNAs were first implicated in immunological pathogenesis, several have been implicated in modulating HIV infectivity and miR‐29s seem particularly interesting.^237^, ^238^, ^239^ Silencing Dicer, and therefore miRNAs, results in an enhancement of HIV‐1 replication, suggesting a role in modulating the immune response to HIV infection.^237^ It is proposed that expression of miR‐29s is directly repressed by interactions with HIV‐1. In fact, miR‐29s are downregulated in the peripheral blood mononuclear cells (PBMC) of HIV‐1 infected patients,^239^, ^240^ but not in “elite suppressors” (patients infected with HIV who maintain low viral load without retroviral treatment), who exhibit comparable PBMC miR‐29s levels to control blood.^240^, ^241^ Furthermore, it has been shown that miR‐29a, induced by IL‐21, directly suppresses replication of the virus via direct interaction with HIV‐1 mRNA, mediating interactions with the RISC complex.^211^, ^237^, ^242^, ^243^, ^244^ miR‐29s are clearly key to understanding immune response to HIV infection.

## 6 CONCLUSION AND PROSPECTIVE

**Figure 2:**
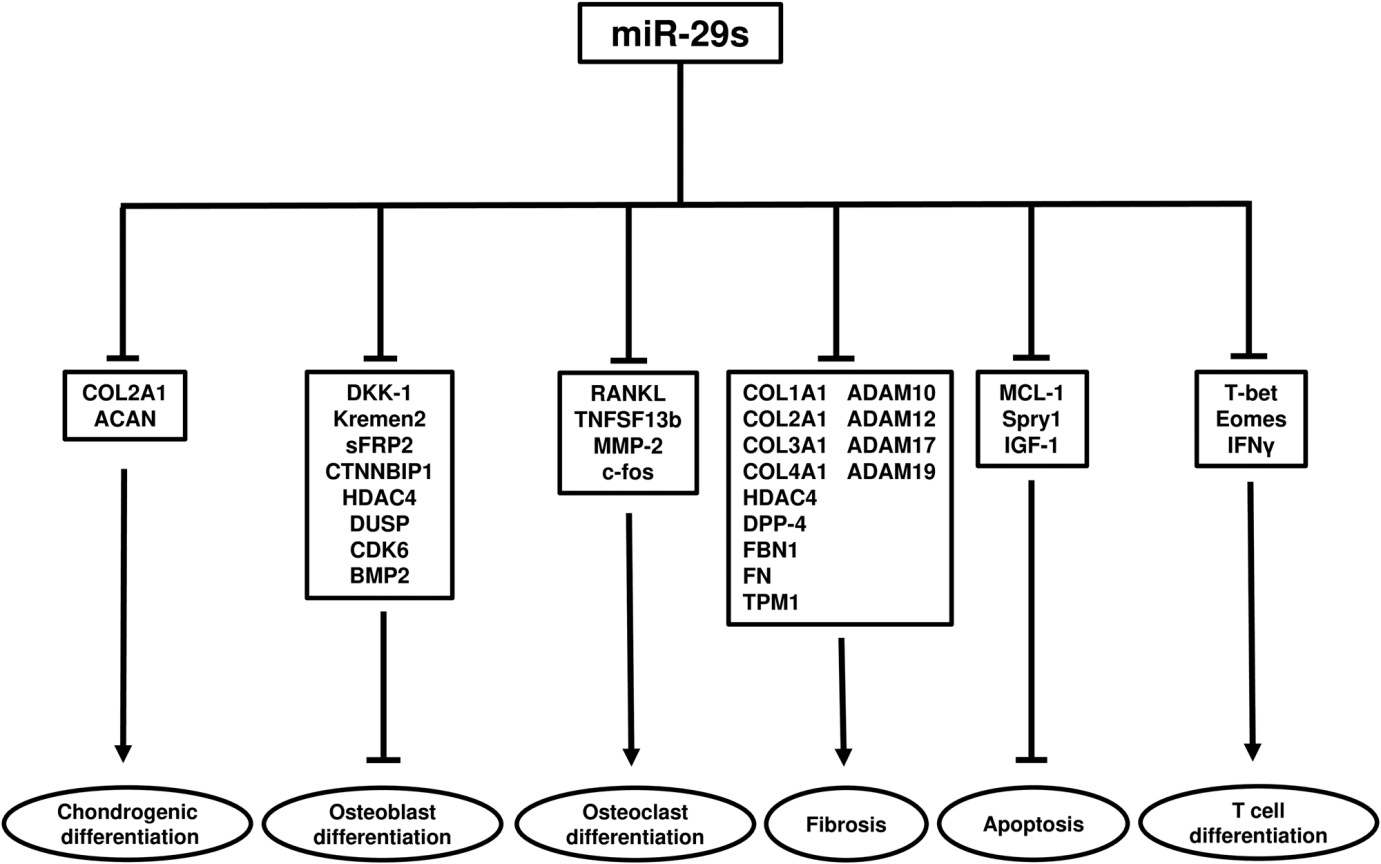
Target genes of miR‐29s in the mechanisms of cell differentiation, fibrosis, and apoptosis. The illustration describes the reported target genes of miR‐29s involved in chondrogenic differentiation, osteoblast differentiation, osteoclast differentiation, fibrosis, apoptosis, and T cell differentiation

In the current review, the miR‐29 family has been shown to promote osteoblast differentiation and apoptosis whilst suppressing chondrogenic differentiation, osteoclast differentiation, fibrosis, and T cell differentiation via the inhibition of target genes (Figure 2). It is clear that miR‐29s could offer therapeutic targets for pathologies involving OA, osteoporosis, cardiorenal disease, and immune disease. However, differing expression levels of miR‐29s in the tissues, cells and serum may need to be taken into consideration (Table 1). In addition to these diseases, further studies have reported that miR‐29s could also provide a promising novel therapeutic approach for reducing excessive collagen deposition in both liver fibrosis and tendinopathy.^245^, ^246^, ^247^ Intravenous administration of miR‐29a has been reported to improve liver fibrosis in mouse models via the downregulation of Col1a1 expression. Watts et al., Similarly, in tendinopathy, intra‐lesional injection of miR‐29a improved the tendon lesion cross‐sectional area in an equine model of collagenase‐induced superficial digital flexor tendon injury via the downregulation of Col3a1 expression.^247^ Millar et al., furthermore, the direct injection of a miR‐29a mimic downregulated the expression of Col3a1 in the tendon of a mouse model of patellar tendon injury.^246^ These preclinical studies suggest that miR‐29a is also an essential negative regulator for liver fibrosis and tendinopathy. Thus, the role of miR‐29s in the etiology of chronic diseases is extensive and a comprehensive understanding of miR‐29s functions may provide a rational for an attractive treatment strategy for disease prevention and/or cure. Further studies are therefore crucial to clarify the precise mechanism of miR‐29s and their potential therapeutic applications.

**Table 1:** Expression of miR‐29s in tissues and serum of humans and animal models of disease

	Osteoarthritis			Osteoporosis			Cardiac disease			Renal disease			Immune disease		
		References	Species		References	Species		References	Species		References	Species		References	Species
miR‐29a	↑	32	Human	ー			↑	164 165 166 168 169 184 185	Human Human Human Rat Mouse Mouse Rat	ー			ー		
	↓	56	Human	↓	33 92 110 113	Mouse Human Mouse Rat	↓	156 157 160 161 167 207	Mouse Mouse Mouse Dog Human Mouse	↓	35 36 154 158 175 192 193 205	Rat, Mouse Mouse Mouse Mouse Rat, Mouse Mouse Mouse Mouse	↓	240	Human
miR‐29b	↑	32 55	Human Human	ー			↑	165 185	Human Rat	ー			↑	209	Human
															
	ー			↓	93	Human	↓	156 157 161 167 207	Human, Mouse Mouse Human, Dog Human Mouse	↓	35 36 154 158 175 190 205	Rat, Mouse Mouse Mouse Mouse Mouse Mouse Mouse	↓	240	Human
miR‐29c	↑	32 57	Human Human	ー			↑	165 185	Human Rat	↑	194 195	Mouse Human	ー		
	ー			ー			↓	155 156 157 160 161 207	Mouse Mouse Mouse Mouse Dog Mouse	↓	35 36 154 158 174 175 205	Rat, Mouse Mouse Mouse Mouse Human, Rat Rat, Mouse Mouse	↓	240	Human

## CONFLICT OF INTERESTS

The authors declare that there are no conflict of interests.
